# Supplementary material for: Mycoplasma bovis Infection Induces Apoptosis Through Gadd45/XIAP in Bovine Macrophages
Source: Microorganisms. 2025 Aug 30;13(9):2031. doi: 10.3390/microorganisms13092031 (PMC12472996; doi:10.3390/microorganisms13092031)
Supplement: Supplementary file 1 [file microorganisms-13-02031-s001.zip › microorganisms-3793085-supplementary.pdf]

Table S1. Primers used in this study

Target	Forward primer (5′-3′)	Reverse primer (5′-3′)
16S rRNA	AAATGACACAAAATGAAGGAAA	AAATGACACAAAATGAAGGAAA
UvrC	AAATGACACAAAATGAAGGAAA	AAATGACACAAAATGAAGGAAA
Bax	ATCATGGGCTGGACATTGGACTTC	CAGCAGGCACTTCAGCGACTC
Bcl-2	TGTGGATGACCGAGTACCTGAACC	GCCAGACTGAGCAGTGCCTTC
β-actin	AGCAAGCAGGAGTACGATGAG	ATCCAACCGACTGCTGTCA
Gadd45	GAGAGCAAAAGACCGAAAGG	GAAGAAAGGCGGGATAGGA
BID	CAGCACGAGAGGACAGAGA	GCAAACACAGAAACACCAAG
RAF	AAGGATGGTAGTTGGAGAAGG	TGAAAGTGAAAAGTGAAAGGG
BAK	TACCAAGGGAGGGAGAAAGG	GGGACAGGGAGAAGGGACT
ENDO-G	CGACACCTTCTACCTGAGCAA	CTCGGCAACTCTCCCATTC
Calpain	CCCAACTCCTCCAAAACCT	GCGTCCTATTCCCACTCCT
Caspase3	TGAATAGAAAGGTGGTGAGGA	CCAGAAACGGGAGGATAAG
FAP1	AAGGAGAGGAAAGGAGTGGT	GCCAACGAGATGTGAGAAG
FAS	CCGTCTTGTTCGCTACATCC	TGCCCCTTTTACCCTTTCAC
ARTS	CCCCTGTGTTGTGGTATTG	TGAGCATTTGTCTGGTGGT
ATM	CACAACCTAATCTGTCCCTTG	TTCTTTCCTTCTCTCTCACCTC
XIAP	ATGAGGTAGAGGGAAATGAAAA	AAATGACACAAAATGAAGGAAA

Table S2. Antibodies used in this study

Antibodies	Source	Identifier
Goat Anti-Rabbit IgG H&L (HRP)	Abcam, UK	ab6721
Beta Actin Polyclonal antibody	Proteintech, USA	20536-1-AP
BAX Polyclonal antibody	Proteintech, USA	50599-2-Ig
Bcl2 Polyclonal antibody	Proteintech, USA	26593-1-AP
GADD45A Polyclonal antibody	Proteintech, USA	13747-1-AP
XIAP Polyclonal antibody	Proteintech, USA	10037-1-Ig

Table S3. SiRNA of Gadd45 and XIAP

SiRNA Name	Sequences(5′ to 3′)
SiGadd45-54	GGAGGAAUUCUCGGCUGGATT
	CCAGCCGAGAAUUCCUCCTT
SiGadd45-169	CGCAUUCAUCACAGUGGAATT
	UUCCACUGUGAUGAAUGCGTT
SiGadd45-65	CGGCUGGAGAGCAAAAGACTT
	GUCUUUUGCUCUCCAGCCGTT
SiGadd45-Negative	UUCUCCGAACGUGUCACGUTT
	ACGUGACACGUUCGGAGAATT
SiXIAP-77	CGAUAAGGAUGAAGAAUUUTT
	AAAUUCUUCAUCCUUAUCGTT
Si XIAP-267	GGAAGACACAGGAGAGUAUTT
	AUACUCUCCUGUGUCUUCCTT
Si XIAP-1269	GUGAAUGCUCAGAAAGAUATT
	UAUCUUUCUGAGCAUUCACTT
Si XIAP- Negative	UUCUCCGAACGUGUCACGUTT
	ACGUGACACGUUCGGAGAATT

Table S4. Geographical distribution of samples

Herd NO.	Locations	Citis	NO. Sample collected	Sample source
1	Eastern region	Hami	12	Nose swab
2	Northern region	Shawan	14	Nose swab and Lung tissue
3		Shihezi	9	Nose swab
4		Yining	11	Nose swab and Lung tissue
5		Gongliu	8	Nose swab
6	Southern region	Kashgar	10	Nose swab
7		Aksu	8	Nose swab
8		Tumxuk	12	Nose swab

Table S5. Bio-chemical identification results

Biochemical identification items	Result
Hydrolysis of gelatin test	Negative
Hydrolysis of arginine test	Negative
Hydrolysis of esculin test	Negative
Decomposition of urea test	Negative
Decomposition of mannose test	Negative
Fermentation of glucose test	Negative
Fermentation of lactose test	Negative
Triphenyltetrazolium chloride reduction test	Positive

Table S6. Results of drug susceptibility test

Types of drugs	Drug Name	Diameter of the inhibition zone/mm	Standard for the Diameter of the Inhibition Zone /nm			Result
			Sensitive	Moderately sensitive	Drug resistance	
Aminoglycoside	Gentamicin	24.35	≥15	13-14	≤12	Sensitive
	Kanamycin	24.35	≥18	14-17	≤13	Sensitive
Tetracyclines	Tetracycline	21.35	≥19	15-18	≤14	Sensitive
	Doxycycline	30.35	≥18	14-17	≤13	Sensitive
Fluoroquinolones	Ciprofloxacin	13.35	≥21	16-20	≤15	Drug resistance
	Ofloxacin	14.35	≥16	13-16	≤12	Moderately sensitive
Quinolones	Norfloxacin	15.35	≥17	13-16	≤12	Moderately sensitive
	Lomefloxacin	17.35	≥22	19-21	≤18	Drug resistance
Nitrofurans, synthetic antibacterial agents	Nitrofurantoin	40.35	≥17	15-16	≤14	Sensitive

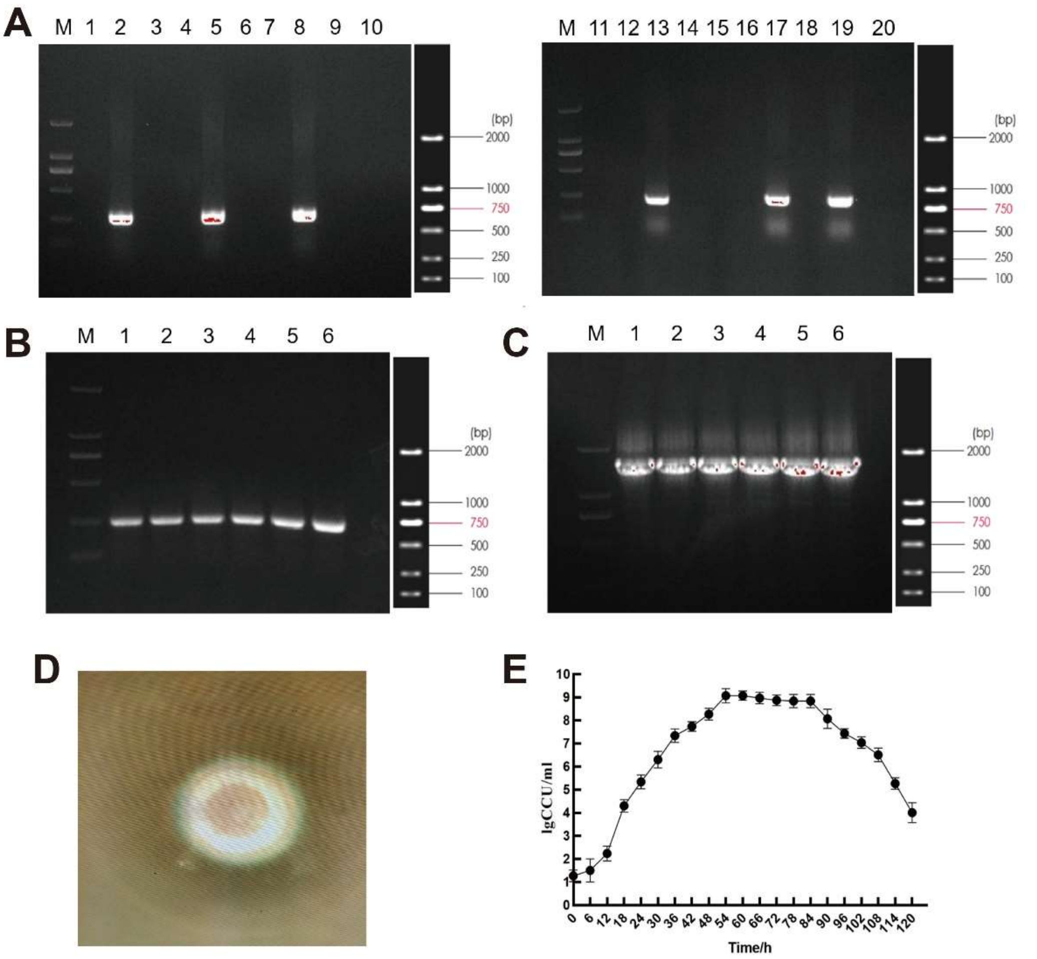

Fig. S1. Molecular identification and growth kinetics of *Mycoplasma bovis* Xinjiang isolate XJ01. (A) Primary screening of clinical specimens: PCR detection of *M. bovis*-specific UvrC gene (238 bp) in 88 clinical samples (84 nasal swabs + 4 tissue biopsies). M: DL2000 DNA Marker; Lanes 1-20: Representative samples. (B) Confirmation of isolates: UvrC PCR validation of six culture-positive isolates. M: DL2000 DNA Marker; Lanes 1-6: Isolates. (C) 16S rRNA gene amplification: 1,500-bp fragment verification of isolates. M: DL2000 DNA Marker; Lanes 1-6: Same as (B). (D) Characteristic colonial morphology: "Fried-egg" colonies on solid PPLO medium (100× magnification). (E) Growth dynamics in liquid medium: XJ01 exhibited logarithmic growth at 12 h, peak titer (1.0×10^9 CCU/mL) at 54 h, and sustained stationary phase from 54–84 h in PPLO broth (data presented as mean ± SD).

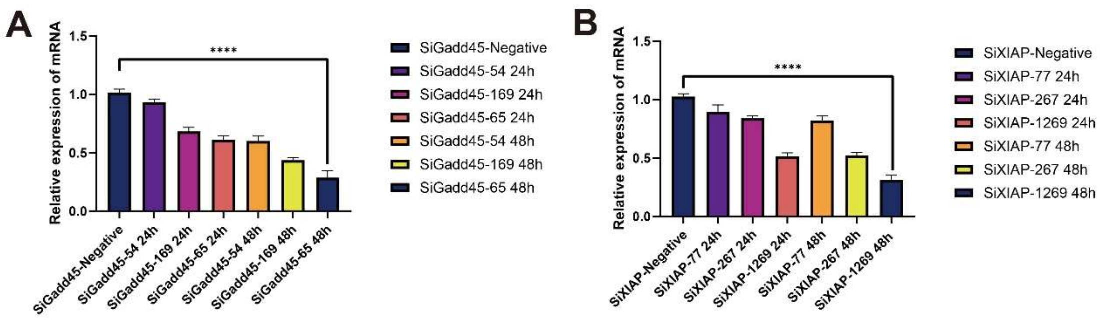

Fig. S2. Screening of effective siRNA fragments targeting Gadd45 and XIAP genes. (A) Gadd45 knockdown efficiency: Relative Gadd45 mRNA levels in BoMac cells transfected with three sequence-specific siRNAs (SiGadd45-54, SiGadd45-169, SiGadd45-65) at 24 h and 48 h post-transfection, demonstrating >70% knockdown efficacy for SiGadd45-169. (B) XIAP silencing validation: XIAP mRNA expression following transfection with XIAP-targeted siRNAs (SiXIAP-77, SiXIAP-267, SiXIAP-1269) at 24 h and 48 h, identifying SiXIAP-1269 as the optimal interfering fragment. All data presented herein represent the results from three separate experiments and are mean ± SD. Ns, not significant ($P > 0.05$), $0.01 < * P < 0.05$, $** P < 0.01$, $*** P < 0.001$, and $**** P < 0.0001$.
